# Public Health Network Structure and Collaboration Effectiveness during the 2015 MERS Outbreak in South Korea: An Institutional Collective Action Framework

**DOI:** 10.3390/ijerph14091064

**Published:** 2017-09-15

**Authors:** KyungWoo Kim, Simon A. Andrew, Kyujin Jung

**Affiliations:** 1 Department of Public Administration, University of North Texas, Denton, TX 76203, USA; kyungwoo.kim@unt.edu (K.W.K.); simon.andrew@unt.edu (S.A.A.); 2 Social Disaster & Safety Management Center, College of Liberal Arts, Korea University, Seoul 02841, Korea

**Keywords:** public health network, collaboration effectiveness, Middle East Respiratory Syndrome (MERS) coronavirus, institutional collective action framework

## Abstract

Following the 2015 Middle East Respiratory Syndrome (MERS) outbreak in South Korea, this research aims to examine the structural effect of public health network explaining collaboration effectiveness, which is defined as joint efforts to improve quality of service provision, cost savings, and coordination. We tested the bonding and bridging effects on collaboration effectiveness during the MERS outbreak response by utilizing an institutional collective action framework. The analysis results of 114 organizations responding during the crisis show a significant association between the bonding effect and the effectiveness of collaboration, as well as a positive association between risk communication in disseminating public health information and the effectiveness of collaboration.

## 1. Introduction

The effectiveness of collaboration reflects a successful implementation of planned actions and procedures to coordinate join activities among seemingly independent set of private and public agencies. An effective collaboration captures not only meaningful interactions previously developed by agencies but also a set of planned actions and procedures contributing to an effective cooperation among various levels of public agencies and private organizations during a crisis [1,2]. An effective collaborative effort can be examined through three dimensions: the expectation that a collaborative effort is contributed by: (1) improved quality of services provision and production, (2) cost savings, and (3) coordination [3,4,5].

However, current literatures on public health emergency response have been particularly silent on the relationship between social positions of organizations and the tangible or intangible advantages that individual organizations can gain from the positions [6]. There are at least two competing perspectives on mechanisms explaining what organizations can expect to gain from their interactions, i.e., as to what type of connections should be sought by organizations in order to reap the advantage of social interactions [7,8].

According to the bonding effect, individual organizations can benefit from their closed-knit social structure when they share risks and gain access to quality information. The bridging perspective suggests public health emergency response requires rapidity and agility of responders to act. The benefits of interaction among first responders are assumed to reduce the cost of response when they can allocate risks through diversification of partners and take advantage of various expertise and skills within their networks [2,6].

This study aims to develop theories to understand a self-organized mechanism to mitigate collective action dilemmas derived from public health network responding to an epidemic event in South Korea, which is known to have a strong hierarchically coordination. The research question guiding this study is what communication strategies governmental agencies take to enhance the effectiveness of intergovernmental collaboration during public health emergency response. This research argues that governmental agencies take strategic communication actions to gain through interactions with other agencies to improve public health service provision. The other argument is that the more positive the opinion an organization has on the flow/channel of information dissemination concerning an epidemic event, the more confident the organization has on the successful collaboration effort. A positive disposition implies favorable perception on successful collaboration even though organizations may have different experiences about how information is shared with the public, which in turns influences the opinion of first responders regarding the effectiveness of collaboration.

This research contributes to a growing body of literature on interorganizational collaboration by focusing on public health emergency response in the arena of communicable diseases. While much of the current research on the application of the Institutional Collective Action (ICA) framework tends to focus on policy arenas in the realm of water conservation, public good markets, and local sustainable policy [1,9,10], this paper examines the advantages of collaboration in the area of public health emergency response. In addition to the application of the ICA framework to the area of public health network, this research extends the application of the framework to an international setting. Although the ICA dilemma is expected to vary across countries [1], the ICA framework has been rarely applied in other governmental settings (except [6,11,12]).

## 2. Institutional Collective Action Framework

Previous research applying the ICA framework explained that, individual organizations do better than other organizations given the nature of their interactions [13,14,15,16,17,18]. Public officials representing their respective organization developed relationships with their partnered organizations. In order to get the work done, they are motivated to seek information on available resources and reached out to individuals across organizational boundaries. According to the ICA framework, through repeated interactions, local governments developed a certain level of trust, commitment, and shared vision. That, interorganizational collaboration is a function of social interactions that can be studied as a social structure. The structure facilitates certain tangible outcomes to an organization (see [1,9]).

However, the ICA framework also posits that intergovernmental collaboration involves issues of coordination, negotiation, and monitoring [1,6,9]. Governmental agencies can encounter an issue of coordination in performing interjurisdictional activities. While coordination is critical for provision of public services requiring interconnected efforts across jurisdictions, the risk of coordination failure can be high if the collective action involves a set of diverse actors and activities. A linkage with a central actor that has critical information may help resolve a problem of incoordination [1].

Moreover, another challenge is that institutional actors may confront bargaining problems or effective monitoring mechanism if they do not have perfect information about other actors’ behaviors [6,9]. Governmental agencies may lack the ability to negotiate division of benefits and costs without knowing about costs each actor is willing to pay for [1]. Each actor has an incentive to reap a greater benefit from the join actions but spend less costs on the join action. If an actor perceived that the other actor does not bear the same burden of the actions, the actor will anticipate higher costs of negotiation and deliberation. Under such situation, the actor will perceive a higher level of uncertainty and thus experiencing difficulties in implementing join activities [9].

Monitoring an agreement is another challenge to intergovernmental cooperation [1,9,19]. Individual actors can show opportunistic behaviors following their interest to contribute less to the join efforts. The risk of opportunism can be higher if partners have limited information about their partners’ behaviors. Without a mechanism to ensuring the credibility of commitment to the collective action, institutional actors may be reluctant to be involved in collaborative efforts. For example, cities in Georgia were less likely to enter interlocal service agreements when the difficulties measuring the services increase beyond a certain point [16]. 

While there are a few empirical studies applying the ICA framework [10,18,20], few have examined ICA problems at the international setting. Even fewer has examined the ICA problems during public health emergency response. Most studies tend to focus on policy arenas in the realm of water conservation, public good markets, and local sustainable policy. In the next section, this paper examines the advantages of collaboration in the area of public health emergency response by integrating Burt’s structural hole theory in testing the ICA framework.

## 3. Collaboration Effectiveness during Public Health Emergency Response: Bonding and Bridging Effect

What explains the effectiveness of collaboration? The effectiveness of collaboration during public health emergency response can be partly affected by access to quality information. Being informed about important developments and situational awareness can save the organization valuable resources, money and the time to act during a crisis [6]. Working closely together as a collective suggests risks are shared among various organizations; and that, the by-product of such densely connected networks provides individuals with group benefits, i.e., they gain access to information and redundancy of shared resources [9].

Densely interconnected individuals—which facilitates the bonding effect—can also generate intangible benefits particularly to an individual organization [7]. Here, the commitment of an individual organization to work jointly during a crisis illustrates the advantage of bonding when risks of inaction by its members can be minimized through sanctions and reputational effects [2]. During public health emergency responses, where risks of failure are shared, effective sanction and monitoring mechanisms become key to guide the collective action, i.e., acceptable and professional behaviors [21]. Reputations of an organization (as well as the collective) are at stake when responding to a crisis suggesting there is a strong incentive for organizations to observe the actions or inaction of another [22,23]. The expectation will motivate each organization to act accordingly in order to maintain their reputation within the network and thus ensuring collaborative efforts are a success [22]. The failure of one affects the ability of others to get the job done. 

However, in the field of public health emergency response, the empirical studies on the effect of bonding have been sketchy. The logistic nightmare aside, the flexibility of relationship requires trust. In presence of a crisis, the risk of failure of individual responders can contribute to the difficulty to make appropriate choices and set priorities. For example, second hand information (especially to evaluate the situation) often leaves out contextual situation influencing outcomes [24]. There are bias about information, which influence the perceived effectiveness of collaboration [25]. For instance, the evaluation of success is shaped by group discussion/interactions [26]. If closed-knit groups have a positive view of the outcome, the likelihood of the individuals in the group to have a positive predisposition about the join efforts would be high. A positive outcome gets repeated because it is appealing to all actors in the networks (rather than trustfulness). According to Burt, a positive image of outcomes can lead to certain individuals to have favorable opinion about outcomes [27].

### 3.1. Network Centrality

The explanations for a network centrality—as a proxy for the bonding effect—on the level of perceived effectiveness of collaboration are as follows: network centrality, which is measured in terms of network size, provides an opportunity for an actor to access information and resources in a timely way from multiple actors and improve their task performance [28,29,30,31]. The focal actor is located at the central location of network can improve its reputation from others and can ensure cooperation from their partners [29]. The prominent actors can access timely information from their partners and better exploit information. The prominent actor also can develop capacity to use information and resources from their partners by expanding expericence of creating and maintaing linkage with other actors [30]. The focal actor that has actual interaction with other actors and has better understandings about the availability of information and resources from their partners, develop innovative solutions, and improve its performance.

A public agency that has linkage with multiple organizations is better able to access core timely information because the focal agency can receive information directly from them [32]. In other words, socialization leads to consistency of opinion. The agency can develop better understanding about a crisis by communicating with multiple organizations and provide better information to their partner agencies. The centralization of the focal agency can improve the effectiveness of the collaborative efforts with other organizations in preventing further crisis to occur.

**Hypothesis** **1.** The higher the level of network centrality of an organization, the higher the perceived level of collaboration effectiveness will be during a crisis.

### 3.2. Network Constraint

The quality of information deteriorates as more intermediaries are involved, however. An organization that is constrained by partners that are directly connected is more likely to receive redundant information from their partners [33]. The actor may not obtain innovative solutions from their homogenous partners. Organizations are constrained if they are positioned within a close-knit network. On the other hand, an organization that exchanges information with other organizations that are connected to each other will gain redundant information rather than novel information, which is essential for identifying the path of the transmission path [34]. The organizations that have high constraints or are densely connected to other organizations may not be able to access new risk information and may not collaborate effectively to prevent the further transmission of infectious disease. In other words, Burt’s logic of network constraint explains that the higher the level of constraints, the lower the perceived level of collaboration effectiveness during the crisis [31].

In the context of interorganizational collaboration, the next best option for the organization is to interact with organizations in other groups. This will allow the organization to gain information that is not available from the closely connected organizations [31,33,34]. For example, Burt suggests that network brokerage enables individuals to access new ideas and develop innovative solutions [35]. He finds that managers that interact with other groups are better able to obtain alternative ideas and develop creative solutions [35]. The managers that arbitrage ideas with their partners that do not talk to each other are more likely to express innovative ideas and received tangible rewards for their performance.

Similarly, a public agency should be able to obtain core information that describes the nature of the problem and invest the given time and resources in responding to the immediate needs [36]. This is important especially when the response agency is able to access information from multiple jurisdictions in responding to a virus outbreak. The agency needs to understand the path of infectious disease transmission, which has a transboundary nature [37]. The agency will also be better able to access the transmission information and improve the effectiveness of interagency collaboration when the agency interacts with organizations in other regions.

**Hypothesis** **2.** The lower the level of constraints, the higher the perceived level of collaboration effectiveness will be during a crisis.

### 3.3. Network Hierarchy

A public agency may choose an efficient mode of communication strategies by exchanging information only with key agencies. Network hierarchy refers to a closed form of network structure organized around a few prominent alters and allows a focal actor to leverage on others through the central actors [31,33,38]. A higher level of network hierarchy means less redundant ties and a more efficient communication ties to access new information.

The argument is a direct criticism of the bonding effect, i.e., when the agency is surrounded with redundant ties, the agency can access novel information [8,33]. However, the bonding effect fails to take into account the idea that an organization may be advantaged if it can communicate with an influential actor within a network, i.e., it is able to access information from all others without investing time and resource. Instead, the organization can create a communication linkage with the higher-level agencies instead of all organizations in the network [39]. The linkage with the central actors, therefore, enables the focal actor to obtain diverse information from the prominent actor that received information from heterogeneous set of other actors and thus allowing the organization to save time and resources.

The network hierarchy captures the logic of the bridging effect. In other words, an organization will have a higher score on a network hierarchy suggests that the organization communicates with a higher-level agency or a prominent agency that already have information from higher level agencies. Interacting with these agencies not only give certain advantages to the organization, i.e., save time and resources for creating and maintaining the linkage with other multiple agencies, but also access to comprehensive information about the crisis. Considering the transboundary nature of an epidemic event, communication with a national or a regional agency will help the public agency at the local level to understand the nature of an epidemic event. Local agencies will be better able to use limited resource for collaborating with other agencies in preventing secondary effect of hazard more efficiently.

**Hypothesis** **3.** The higher the level of network hierarchy, the higher the perceived level of collaboration effectiveness during the crisis.

### 3.4. Social Media and Collaboration

How does effective dissemination of information affect interorganizational collaboration? The ability of various organizations to disseminate information regarding a crisis also creates conditions that can promote interorganizational collaboration during responses [40,41]. Collaboration among public agencies facilitates a culture of responsiveness among first responders and responsible agencies. Shared values and concerns for the public are important elements during public health emergency response [42]. Such a culture provides a condition that allows a highly productive team to work together in order to develop a cohesive response.

Scholars have argued that online information channels can expand ways to disseminate timely information to the target population as a new epidemic situation develops [43,44,45,46,47]. Social media also enables members of the public to communicate their situations easily with government agencies and improve situational awareness of public health emergency response organizations [45]. Social media is also powerful means to involve members of the public in volunteering and donation for disaster response [44]. For instance, following the Red River flooding of 2009, a Facebook group helped the city of Fargo (ND, USA) to recruit 5 percent of the total population when the city suffered from low response capabilities.

The literature on risk communication provides clues on the mechanisms to enhance the effectiveness of intergovernmental collaboration. Risk communication refers to an action of “exchanging information about health and/or environments between interested parties” [47]. Such information includes not only factors of health and environment risk itself but also government decision for controlling and managing risks. Effective risk communication is critical for informing the public of the very nature of hazards and governmental actions to avoid inaccurate perception of the hazard risk and unnecessary sufferings from the lack of knowledge [48]. Timely and reciprocal information provision can improve trust on governmental actions and help them follow guidance from governmental agencies while high levels of trust in governmental actions can discourage individuals to take personal protections [43,49,50].

**Hypothesis** **4.** The more an organization employed the social media to disseminate information to the public, the higher the level of perceived effectiveness of interorganizational collaboration.

## 4. The 2015 Middle East Respiratory Syndrome (MERS) Outbreak in South Korea

South Korea suffered from a MERS outbreak in 2015. One argument suggests that the country failed to control the transmission of the infectious disease from the first patient, who travelled internationally to the Middle East. MERS was a novel pathogen at the time and most cases had been found in Saudi Arabia [51]. The disease is caused by a virus called by MERS Coronavirus, and the common symptoms include shortness of breath, cough, and fever [52,53]. Three in ten infected patients tend to die. Although the virus is thought to be transmitted through close contact with infected persons, there is no precise understanding of the mode(s) of transmission. There is currently no recommended vaccine or specific treatment for the virus infection. Most medical doctors and the national public health authority did not have knowledge about the nature or transmissibility of the virus. The first-MERS patient who had traveled to Middle East found that he had fever and visited five hospitals but could not find the cause of the symptom in the first four hospitals [54]. Eventually, the last hospital he visited identified that he had become infected with MERS. The national public health authority was not also effective in controlling the transmission of infectious disease from the confirmation of the first case on 20 May 2015. Epidemic investigators from the South Korea Center for Disease Control (CDC) set a narrow quarantine range and missed other clinicians and family members who had come into contact with the patient [55].

The failure of the initial response led to further transmission that resulted in 186 confirmed cases, the death of 38 and the quarantine of about 17,000 people [56]. The mortality rate was the second highest in the world. People who were under quarantine were to stay at home for fourteen days to prevent potential transmission to others. The outbreak came under control on 15 June 2015 and no further transmission was declared on 27 July 2015 [57]. In addition, the outbreak had other societal impacts, such as the disruption of education and other economic activities. When there was great anxiety of infection among people, about two thousand schools canceled the classes and people avoided visiting hospitals, shopping malls, and amusement parks [58]. Not only the high-risk patients and the isolated resident but also parents who had children with cancelled classes had difficulties to continue their existing economic activities [59]. The outbreak, especially, damaged tourism industries and the country had two million tourists less than expected during the year of the outbreak [60].

Public agencies had several challenges in seeking risk information to prevent the further transmission during the outbreak response. The national public health authority disclosed information about hospitals with MERS cases two weeks after the first patient was identified and was blamed for the further transmission [61]. Residents who had not known about the hospitals and visited the facilities became infected. The Seoul metropolitan government criticized the national government regarding the transparency regarding the hospitals with MERS infection [62]. Public health agencies also had difficulties in using the National Public Health Information System, which was supposed to facilitate the exchange of information among agencies. Because the information system did not allow local public health agencies to input their local risk information instantly, the system was not able to integrate the local information and failed to provide up-to-date transmission information for the users [63]. It is evident that South Korea public agencies were desperate to gain information about MERS in order to prevent the further transmission of infectious disease in their jurisdictions. The MERS crisis reflects challenges in interorganizational collaboration, especially the effectiveness of collaboration among public agencies. Along with information sharing, national, regional, and local public health agencies had challenges in cooperation for resource exchange. For instance, one local public health agency of Seoul requested equipment (e.g., ambulance) and personnel to the regional agency to tackle with high risk patients in the local jurisdiction but the upper level agency did not respond to the low-level agency’s demand immediately. The slow response was not beneficial for governmental agencies to prevent further transmission of the infectious disease [55].

## 5. Research Design and Methods

### 5.1. Data

The data were collected in the Seoul metropolitan region and 25 other local jurisdictions with reported MERS cases in February 2016. The survey was administered to three national ministries, 10 regional governments/agencies, 50 local public health agencies, 58 local police agencies, and 48 local fire agencies between January and March 2016. The regional and local agencies represent Seoul Metropolitan areas and 25 other local areas that had hospitals with MERS cases. All local public agencies in the Seoul Metropolitan areas were selected because 64 percent of local jurisdictions had more than one MERS case and residents have high mobility within the region.

Local agencies in non-Seoul metropolitan areas were identified from a list of affected areas from the Ministry of Welfare and Health. We received complete responses from 120 local agencies responding to the crisis including three regional agencies, 38 local public health agencies, 47 police agencies, and 32 local fire agencies. The response rate was 71 percent. Local jurisdictions with the responding local public health agencies do not have significant differences with non-responding agencies in term of socio-demographic characteristics, such as population density and elderly population (see Table 1). Although only 120 public agencies provided completed surveys, we utilized 19 other governmental agencies that provided only network information but not complete information, including three national agencies and one regional agency. Their responses were used to provide network information in order to construct the risk communication network.

### 5.2. Variables

#### 5.2.1. Interagency Collaboration Effectiveness 

We measured interagency collaboration effectiveness based on three survey items: improved quality of services provision and production, costs savings, and coordination. Public officials from the selected agencies were asked to judge interagency effectiveness in the tree dimensions using the following survey items:

Quality of service and production: “Please rate how much did interagency collaboration help your agency improve the service quality to address the needs of high risk patients and suspected residents during the first month of the outbreak response (20 May–14 June 2015)?”

Cost savings: “Please rate how much did interagency collaboration help your agency to save costs regarding service quality to address the needs of high risk patients and suspected residents during the first month of the outbreak response (20 May–14 June 2015)?”

Coordination: “Please rate how much did interagency collaboration help your agency to coordinate efforts to address the needs of high risk patients and suspected residents during the first month of the outbreak response (20 May–14 June 2015)?”

The response on each item is rating of each dimension and ranges from 0 (not at all) to 10 (very much helpful). The response categories are ratings of the responses and are summed as one index variable (Cronbach’s alpha = 0.9102). As shown by the descriptive statistics in Table 2, the average value is 21.44 and the standard error is 6.65.

Although there are various ways to measure interorganizational collaboration, we used two survey items asking the following question to measure collaboration:

Sending information: “To which organization did your organization send information regarding MERS during the outbreak directly (information about high risk patient, hospital, or response information).”

Receiving information: “To which organization did your organization receive information regarding MERS during the outbreak directly (information about high risk patient, hospital, or response information). “

The data were managed as a non-directed matrix after we merged the survey response in the same direction to make the most use of information about interagency communication. Social Network Analysis was employed to analyze the data to determine interorganizational communication linkage [2,6,12]. Table 3 presents a summary of the network statistics. The number of agencies in the network is 154, with ties of 425. On average, each agency has 2.76 ties with other agencies. The average length of the shortest path is 2.97 and the distance-based cohesion is 0.31.

#### 5.2.2. Network Centrality

The network centrality is operationalized by calculating the normalized degree centrality score, which is the number of nodes adjacent to the focal node (i.e., degree) [64,65]. The literature used degree centrality to measure the prominence of an actor in an interorganizational network. UCINET 6 (version 6.012, Analytic Technologies, Harvard, MA, USA) was employed to calculate the normalized score, which is degree divided by maximum possible number of nodes [64].

#### 5.2.3. Network Constraints

Network constraint is used to measure bridging effects that allow the focal actor to access heterogeneous information through directly connected alters [66]. Network constraint is the extent to which the focal node is connected to others that are connected to each other [29,35]. A high level of network constraint means few structure holes and decreases the likelihood of a local bridging role between directly connected alters while a low level of the constraint means many structure holes increase the likelihood of local bridging role. UCINET 6 was employed to calculate the constraint score based on the undirected matrix [64].

#### 5.2.4. Network Hierarchy

Network hierarchy measured as the extent which total constraints are located at the focal node’s single other [33]. When an actor has a high hierarchy score, the actor has an alter that interact with multiple partners and can help the actor to access new information efficiently [39]. We calculated the hierarchy score using UCINET 6 [64].

#### 5.2.5. Online Channel Effectiveness

We measured the online channel effectiveness as an index summing the response to the three survey items rating the effectiveness of the three types of online channel to disseminate risk information to the public: agency website, social media page (e.g., Facebook, Twitter), and agency blogs. The response categories range from 0 (not used at all) to 10 (very much effective). The summative index has high reliability (Cronbach alpha = 0.990).

A set of control variables was also included at the organizational level, i.e., organizational types and socio-demographic characteristics of community served by the organizations. First, two dummy variables for organizational types were created for a local police agency and fire agencies, which support local public health agencies, regional and national governmental agencies. It was expected that the two types of local agencies may have differences with governmental agencies that led the outbreak response. We also added two socio-demographic variables: population density and elderly population. Population density was measured as the total population divided by the land area (km^2^) and elderly population was measured as the proportion of elderly population to total population. It was also expected that local agencies that served densely populated area or a large proportion of senior population will face challenges when responding to the outbreak because citizens are more vulnerable to the virus transmission via contact.

## 6. Methods of Analysis

An ordinary least square (OLS) regressions was used to test the effects of the three types of communication strategies and online channel on interagency collaboration effectiveness. OLS regressions were applied to similar research that examine the relationship between network effects and outcomes [2,6,12]. Six models was estimated including three models for all agencies and three other models for local public health agencies. Although all the models do not have multicollinearity issues (average variance inflation factor < 2), Breusch-Pagan tests show that the first models had issues of heteroscedasticity. We employed robust regressions to address the problem in estimating the first three models. 

## 7. Results and Discussions

Table 4 and Table 5 present the OLS regression results testing the effects of network position and communication technology on the effectiveness of interagency collaboration. Table 4 is testing for all types of organization and Table 5 is for local public health agencies. The regression results do not support the first hypothesis that the greater the network centrality, the higher the perceived level of collaboration effectiveness during the crisis. Although the coefficients of the variable are positive (0.362 and 0.146) in the Models 1 and 4, network centrality does not have a significant relationship with the effectiveness of interagency collaboration at the level of 0.1. The benefits of accessing essential risk information directly from multiple agencies may not outweigh the costs of creating and maintaining linkages with diverse agencies during the outbreak response. Especially, different types of public agencies have different culture and information standards, which create challenges in expanding communication with multiple agencies [67,68]. An interview with one local public health official supports the quantitative findings:

“One of the challenge for interagency collaboration was a difference in information standards across different types of agencies. My local public health agency had confusion in sharing information about period and termination date of quarantines with police or fire agencies because the supporting agencies did not have the same information standard for reporting the quarantine start and termination dates.”

Model 2 results confirm the second hypothesis that the lower the level of constraints, the higher the perceived level of collaboration effectiveness during the crisis (Table 4). The coefficient of network constraint is −6.862 and is significant at the levels of 0.01 in Model 2. A public agency that communicates with agencies in other regions can have a better understanding of the infectious disease transmission and may be better able to collaborate with other agencies to address the immediate response needs. High mobility of residents may increase the importance of communication across regions to accurately identify the transmission path to prevent further transmission in their jurisdictions. For instance, two residents lived in a region about 250 miles away from the hospital with tens of MERS cases and the two of them became infected. On the other hand, Model 5 does not support the hypothesized effects of network constraints on perceived levels of interagency collaboration effectiveness. Although the coefficient of network constraint is −0.038 in the model, the coefficient is not significant at the level of 0.1. Most local public health agencies that led the outbreak response in their regions communicated with agencies in other jurisdictions or high-level agencies, so network constraints do not have differentiated effects on the levels of interagency collaboration effectiveness.

Model 3 result does not support the third hypothesis that the higher the level of network hierarchy, the higher the perceived level of collaboration effectiveness during the crisis. Although the coefficient of network hierarchy is positive (β = 0.615), the coefficient is not statistically significant at the level of 0.1. Communication with a few prominent agencies may not necessarily help public agencies better understand the nature of the outbreak and improve the effectiveness of interagency collaboration effectiveness. On the other hand, Model 6 confirms the hypothesized effects of network hierarchy on interagency collaboration effectiveness among local public health agencies. The coefficient of network hierarchy is 13.53 and significant at the level of 0.05. When local public health agencies that represent their local jurisdictions communicate with national, regional, or other prominent agencies have better understanding about the infectious disease transmission and can develop better strategies to coordinate interagency efforts in preventing the further transmission in the jurisdictions.

The first three model results support the fourth hypothesis that risk communication is positively associated with the perceived levels of interagency collaboration effectiveness. The coefficients of the three models are each 0.042, 0.046, and 0.043 and are statistically significant at the level of 0.01. A public agency that used internet, social media and blogs can expand their communication channels to inform the public about the outbreak. They can respond and improve interagency efforts to prevent further transmission of the virus within their local jurisdictions. Online communication channels also helped improve the awareness of the public and contribute to interorganizational efforts toward the prevention of the transmission.

Additionally, the results of the model show that organizations, locations, and demographic characteristics of local organizations do not have a significant effect on the perceived levels of interagency collaboration effectiveness. Although local police agencies or fire agencies tend to have a lower level of perceived interagency collaboration effectiveness than other agencies, most coefficients in the models are not statistically significant at the level of 0.1. Elderly population served by local agencies has a positive effect on the dependent variable but the coefficient is not statistically significant. An agency serving densely populated residents, on average, reported to have favorable view on interagency collaboration (Model 6). This finding is unexpected, i.e., public health agencies with populous local jurisdictions tend to have high levels of interagency collaboration effectiveness. Public agencies in the populous jurisdictions may be sensitive to potential widespread transmission and choose to collaborate better with other agencies than those jurisdictions that are serving less densely populated residents.

## 8. Conclusions

This study suggests that interagency risk communication strategies and online risk communication are important means to improve interagency effort toward a virus outbreak. Public agencies need to take adequate interagency risk communication strategies to access core information to prevent the transmission of an infectious disease that has a transboundary nature. Bridging strategies are useful for public agencies to access new risk information from other regions, understand the dissemination path of the disease, and exert better collective efforts toward the prevention of transmission. Efficiency-based risk communication helps local public health agencies to obtain critical risk information and save time and resource for productive activities to prevent the further transmission of an infectious disease. Finally, when public agencies use online media, such as websites and social media, the agencies can expand the channel of public risk communication. The expanded communication channels help improve the public awareness of the outbreak response, individual protection actions, and contribute to interagency collaboration. 

The findings have several practical implications for effective interagency communication and collaboration in response to epidemics that has a transboundary nature. First, public agencies should be able to exchange risk information with agencies in other regions or jurisdictions. Bridging strategies enable a public agency to access information, which is not available within or nearby local jurisdictions to understand better the path of infectious disease transmission. When the agency can receive risk information from other regions where their residents travel frequently, the agency can identify the path of transmission to their residents and take response actions to prevent further transmission.

Second, communication with a national, regional agency, or other prominent agency can help a public health agency to save time and resource for improving the outbreak response in the jurisdictions. Although a public agency that leads the outbreak response in their local jurisdiction can create linkages with multiple linkage with other agencies and obtain information from them, the agency should divert resource and time for the actual response to prevent the further transmission to their local population. Instead of creating multiple communication linkage, communication with the prominent agencies that exchange information with other regional or local agencies can obtain comprehensive information about the transmission path.

Finally, expansion of communication channel is helpful for a public agency to raise the situational awareness of the public regarding local response status and improve interagency efforts to prevent the further transmission of the infectious disease. Online media, such as websites, blogs, and other social media allows the public agency to disseminate up-to-date risk information to residents and help residents to understand how the individuals can take protective actions and contribute to interagency efforts toward the control of the transmission. When members of the public as co-producer of public services have better understandings of the response [69], they can be better able to contribute to the control of the transmission.

The study has several limitations in testing the effects of the three communication strategies and online channel use on intergovernmental collaboration effectiveness. First, the study does not use objective measures to assess the outcome variables. Because there is no archival data that assess agency or collaboration performance, this study used the perception of public officials who are knowledgeable about their agency response. Even though it is hard to establish objective indicators for collaboration performance, future studies may develop objective measures for public health emergencies and compare the measure with the subjective measures. Second, the study might not be generalizable to all regions or local areas. This study focused on Seoul metropolitan areas and other local areas that had MERS cases due to time and costs. This study did not investigate other local areas or regions that were affected by the outbreak although the areas did not have confirmed cases.

## Figures and Tables

**Table 1:** Differences between responding jurisdictions and non-responding jurisdictions.

Characteristics	Responding	Not Responding	*p*-Value *
N	38	12	
Population density (#/km^2^)	9450.4679	13,937.7	0.388
Senior population (%)	12.61	12.46	0.266

Note: * Independent *t*-test results.

**Table 2:** Descriptive statistics.

Variables	N	Mean	Std. Dev.	Min	Max
Collaboration effectiveness	112	21.44	6.65	0	30
Network Centrality (degree)	112	2.17	1.82	0.65	15.03
Network Constraint	112	0.75	0.30	0.08	1.39
Network Hierarchy	112	0.23	0.34	0	1
Fire	112	0.24	0.43	0	1
Police	112	0.39	0.49	0	1
Location (Seoul)	112	0.49	0.50	0	1
Population density (km^2^)	38	9450.47	8529.01	61.04	28,300.43
Elderly population	38	0.13	0.04	0.07	0.26

**Table 3:** Summary of network statistics.

Attributes	Value
Number of organizations in networks	154
Number of ties	425
Collaborators per organization (average degree)	2.76
Average distance (among reachable pairs)	2.97
Distance-based cohesion	0.31

**Table 4:** Explaining collaboration effectivenesss (all cases).

Explaining Collaboration Effectiveness	Model 1	Model 2	Model 3
*Network Effects:*			
Network Centrality (Degree)	0.362		
	(0.289)		
Network Constraint		−6.862 ***	
		(2.278)	
Network Hierarchy			0.615
			(2.166)
Communication Channels Effectiveness (Dissemination via Internet, Blog, Social Media)	0.042 ***	0.046 ***	0.043 ***
	(0.008)	(0.008)	(0.009)
*Organizational Types:*			
Police	−0.879	−0.114	−1.738
	(1.563)	(1.391)	(1.371)
Fire	−2.424	−0.359	−3.171 *
	(1.914)	(1.990)	(1.815)
Location (Seoul)	1.583	0.643	1.820
	(1.250)	(1.186)	(1.259)
Constant	20.155 ***	25.686 ***	21.191
	(1.593)	(1.626)	(1.187)
N	112	112	112
F-Value	6.42 ***	7.79 ***	6.25 ***
R-Squared	0.081	0.134	0.075

Note: *** *p* < 0.01; * *p* < 0.1; Robust standard errors in parentheses.

**Table 5:** Explaining collaboration effectiveness (local agencies only).

Explaining Collaboration Effectiveness	Model 4	Model 5	Model 6
*Network Effects*:			
Network Centrality (Degree)	0.146		
	(0.638)		
Network Constraint		−0.038	
		(5.492)	
Network Hierarchy			13.532 **
			(5.700)
Communication Channels Effectiveness (Dissemination via Internet, Blog, Social Media)	0.2	0.199	0.215
	(0.145)	(0.146)	(0.134)
Population Density	0.000	0.000	0.000 *
	(0.000)	(0.000)	(0.000)
Elderly Population	28.381	27.689	33.503
	(23.855)	(23.791)	(22.014)
Constant	13.595 ***	14.127 **	10.767 **
	(4.819)	(5.379)	(4.194)
N	38	38	38
F-Value	1.24	1.22	2.84 **
R-Squared	0.1303	0.1289	0.256

Note: *** *p* < 0.01; ** *p* < 0.05; * *p* < 0.1; Robust standard errors in parentheses.
